# When species matches are unavailable are DNA barcodes correctly assigned to higher taxa? An assessment using sphingid moths

**DOI:** 10.1186/1472-6785-11-18

**Published:** 2011-08-01

**Authors:** John James Wilson, Rodolphe Rougerie, Justin Schonfeld, Daniel H Janzen, Winnie Hallwachs, Mehrdad Hajibabaei, Ian J Kitching, Jean Haxaire, Paul DN Hebert

**Affiliations:** 1 Department of Integrative Biology & Biodiversity Institute of Ontario, University of Guelph, Guelph, ON, N1G 2W1, Canada; 2 Department of Biology, University of Pennsylvania, Philadelphia, PA 19104, USA; 3 Department of Entomology, Natural History Museum, London, SW7 5BD, UK; 4 Honorary Attaché, Muséum National d'Histoire Naturelle de Paris, Le Roc, F-47310 Laplume, France; 5 ECODIV, Université de Rouen, Bâtiment IRESE A, Place Emile Blondel, F-76821 Mont Saint Aignan Cedex, France

## Abstract

**Background:**

When a specimen belongs to a species not yet represented in DNA barcode reference libraries there is disagreement over the effectiveness of using sequence comparisons to assign the query accurately to a higher taxon. Library completeness and the assignment criteria used have been proposed as critical factors affecting the accuracy of such assignments but have not been thoroughly investigated. We explored the accuracy of assignments to genus, tribe and subfamily in the Sphingidae, using the almost complete global DNA barcode reference library (1095 species) available for this family. Costa Rican sphingids (118 species), a well-documented, diverse subset of the family, with each of the tribes and subfamilies represented were used as queries. We simulated libraries with different levels of completeness (10-100% of the available species), and recorded assignments (positive or ambiguous) and their accuracy (true or false) under six criteria.

**Results:**

A liberal tree-based criterion assigned 83% of queries accurately to genus, 74% to tribe and 90% to subfamily, compared to a strict tree-based criterion, which assigned 75% of queries accurately to genus, 66% to tribe and 84% to subfamily, with a library containing 100% of available species (but excluding the species of the query). The greater number of true positives delivered by more relaxed criteria was negatively balanced by the occurrence of more false positives. This effect was most sharply observed with libraries of the lowest completeness where, for example at the genus level, 32% of assignments were false positives with the liberal criterion versus < 1% when using the strict. We observed little difference (< 8% using the liberal criterion) however, in the overall accuracy of the assignments between the lowest and highest levels of library completeness at the tribe and subfamily level.

**Conclusions:**

Our results suggest that when using a strict tree-based criterion for higher taxon assignment with DNA barcodes, the likelihood of assigning a query a genus name incorrectly is very low, if a genus name is provided it has a high likelihood of being accurate, and if no genus match is available the query can nevertheless be assigned to a subfamily with high accuracy regardless of library completeness. DNA barcoding often correctly assigned sphingid moths to higher taxa when species matches were unavailable, suggesting that barcode reference libraries can be useful for higher taxon assignments long before they achieve complete species coverage.

## Background

Taxonomic assignments are crucial for effective communication of biological research, enabling comparability between studies. Yet, the ability to categorize biodiversity effectively and accurately is hampered by a lack of taxonomic experts [1]. DNA barcoding has been proposed as a method capable of partially alleviating this "taxonomic impediment" by enabling accurate species identifications by non-specialists using nucleotide comparisons across a standard gene region [2].

In a typical scenario, a specimen of unknown species affinity is encountered, the DNA barcode of the query is sequenced and then compared with a reference library of DNA barcodes [3] to establish a species match for the query. However, just as morphological identification keys cannot provide accurate binomial names for queries from species not included in the key, DNA barcoding cannot assign a species identification when there are no barcode records for conspecifics in the reference library. Consequently, barcoding appraisal studies usually require *a priori *knowledge that the species of the query is present in the reference library (e.g. [4-6]). In real life, a consequence of widespread routine use of DNA barcoding is that failed species matches (e.g. < 98% similarity with the closest library sequence [5]) are frequently encountered (e.g. [7]). In such situations it may be tempting to attempt assignment to a higher taxonomic level (i.e. genus, tribe, subfamily). For example, Armstrong and Ball [8] suggested their query barcode sharing 94.6% similarity with the closest library match (*Clostera albostigma*) was a likely congener but not conspecific of the reference library barcode. There is considerable disagreement over the likely accuracy and appropriateness of such assignment attempts (e.g. [1,5,9-11]), which is not surprising given the different purposes and criteria employed.

### DNA barcoding assignment to higher taxa

Hebert et al. [12] expressed optimism for barcode-based assignments to higher taxa in animals. Such assignments are useful as shorthand for phylogenetic hypotheses from which biological characteristics of organisms can be predicted. For example, by assigning a specimen to the genus *Aellopos *one can predict that as a caterpillar it most likely fed on plants of the family Rubiaceae [13]. The capacity to make predictions based on taxon membership is especially pertinent where fundamental impediments, e.g. an egg or an incomplete specimen, preclude morphology-based detection of characteristics. While assignment to pre-determined taxa is an operation distinct from the description of taxa, assignment accuracy is related to the ability of the character system used as the basis of assignment to track organismal phylogeny (i.e. display a phylogenetic signal [14]). This operation is confounded by the fact that many currently recognized supraspecific taxa are not natural [10]. In such cases, the failure of a character system to provide accurate assignments can reflect "imperfect" taxonomy rather than the lack of phylogenetic signal.

**Figure 1:**
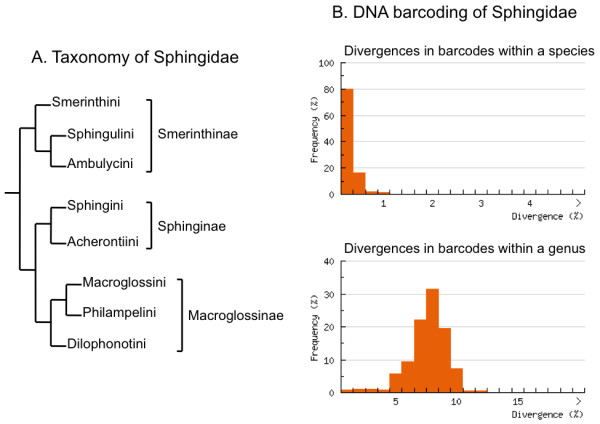
**Taxonomy and DNA barcoding of Sphingidae**. A). A tree representing the taxonomy of Sphingidae showing the three subfamilies and eight tribes currently recognised (based on the classification and relationships in Kitching and Cadiou [16]) and used for the purposes of evaluating assignment success in our experiments. There are 202 currently recognised genera. B) DNA barcoding of Sphingidae. The graphs show the divergences in DNA barcodes within sphingid species and between sphingid species within the same genus. This is based on the publicly available sphingid DNA barcodes on BOLD. A "barcode gap" between the intra and interspecific divergences indicates the ease of species assignment based on DNA barcodes and a "best match" type distance criterion.

In this study, we test the ability of DNA barcodes to enable accurate higher taxon assignments. Specifically we ask: If species coverage in the DNA barcode library is incomplete, can the barcode from a sphingid species not represented in the library be assigned to the genus it belongs to, or, recognised as being from a sphingid genus missing from the library? Likewise, can the barcode from a sphingid genus not represented in the library be accurately assigned at the tribe and subfamily level? We address these questions using the moth family Sphingidae because a comprehensive global reference barcode library is available (86% of known species [15]) containing relatively stable and well-studied taxa (Figure 1A). This enables us to assemble sub-libraries with a wide range of different species completeness and also provides a robust taxonomic framework against which to judge assignment accuracy. We evaluated assignment accuracy using concordance with the current classification of Sphingidae [16] while recognising that morphologically derived taxonomy represents falsifiable hypotheses. Consequently, we also examined the assignments *a posteriori *in light of a more recent phylogenetic study of the family [17]. Sphingidae is the target of a global barcoding campaign [15] and shows high success for species-level barcode identifications (Figure 1B).

### Assignment criteria

Since Hebert et al. [12] proposed that DNA barcoding could be used to assign queries to higher taxa, researchers have performed higher taxa assignments using *ad hoc *criteria based on the frequency of best hits, degree of sequence similarity, bootstrapping or BLAST scores (e.g. [18-22]). However, these studies usually involved fragmentary tissues of unknown taxonomic origin and consequently assignments could not be independently confirmed (i.e. using morphology). Therefore, both the accuracy and optimal approach for such assignments remain unclear. In this study, we test the extent to which assignment accuracy depends on assignment criteria applied by comparing the performance of several approaches employed in prior studies.

### Tree-based assignment criteria

While some consider the use of tree-based assignment approaches controversial [23], we consider it justified for supraspecific taxa sharing phylogenetic as opposed to tokogenetic affinities. Using tree-based criteria, queries are successfully assigned when they cluster with barcodes from their correct taxon [24]. Meier et al. [25] use the following example where they imagine a reference library containing a chimp barcode but no human barcode to illustrate the difficulty with such an approach: "Imagine a query clustering with a chimp barcode. Based on the query's position, one cannot decide whether it comes from *Homo sapiens *or another chimp, i.e., forming a cluster on a tree is logically insufficient for assigning a sequence." We address this concern by establishing objective rule sets for our tree-based assignment criteria based on topology (Table 1). We include assignment criteria that require a taxon to be "monophyletic" or "exclusive" for a query to be assigned to that taxon (Table 1). This requires that we overlook the fact that trees based on COI do not perfectly track organismal phylogeny at deeper levels [14] and that many "traditional" taxa are not monophyletic [17]. Ekrem et al. [9] suggest the inability of COI analysis to reconstruct monophyletic taxa prohibits the use of barcodes for higher taxon assignments.

**Table 1:** Overview of assignment criteria used in this study

Criteria^a^	Requirements for "Positive" Assignment of Q (query)^b, c^
"Liberal"	Q is sister to a single member of a taxon, (*Aus aus*, Q), or a clade of members of a single taxon, ((*Aus aus, Aus bus*)Q), the assignment is that of the taxon *Aus*.

"Strict"	Q is nested within a clade comprising of members of a single taxon, (*Aus aus*, Q)*Aus bus*), the assignment is that of the taxon *Aus*.

"Liberal & exclusive"	Q is sister to a single member of a taxon, (*Aus bus*, Q), or a clade of members of a single taxon, ((*Aus aus, Aus bus*)Q), and members of taxon, *Aus*, are not found elsewhere on the tree except in an *Aus*+Q clade, the assignment is that of the taxon *Aus*.

"Strict & exclusive"	Q is nested within a clade of a single taxon, ((*Aus aus*, Q)*Aus bus*), and members of *Aus *are not found elsewhere on the tree except in an *Aus*+Q clade, the assignment is that of the taxon *Aus*.

"Best match"	Q is simply assigned to the genus of the most similar library sequence based on K2P distance.

"Best close match"	Q is assigned to the genus of the most similar library sequence based on K2P distance provided it falls below a threshold value.

^a ^The first four criteria are tree-based criteria, the last two are non-tree-based criteria.

^b ^When the requirements for a positive assignment were not met, then Q was unassignable (i.e. "ambiguous").

^c ^The examples given refer to genus assignments but were extended to tribe and subfamily using the exact same requirements.

Previous barcoding studies employed neighbor-joining (NJ) algorithms [26] to produce "Taxon ID trees" since the goal of DNA barcoding is species assignment and species discovery and not phylogenetic reconstruction [6]. In this study we used NJ as an approximation to phylogenetic analysis due to computational constraints and the large number of replications undertaken. NJ provides additional comparability as both BOLD [3] and GenBank [27] use NJ in their tree-based identification options. Our tree-based assignment criteria are equally applicable regardless of tree construction method although use of trees selected with a different optimality criterion may produce different results.

### Direct sequence comparison assignment criteria

In addition to tree-based assignment we used criteria based on direct sequence comparison. We chose not to consider "character-based" approaches (e.g. [28]) because nucleotide synapomorphies are unlikely to be pure (i.e. consistency index = 1 [10]) and compound diagnostics have proven unwieldy [29-31]. Of the two assignment criteria we use, both based on K2P [32] genetic distance (Table 1), the least stringent is "best match". A query is assigned the taxon of the reference barcode that it most closely matches irrespective of how similar the query and library barcodes are. Under this criterion some false assignments are inevitable. A "false-positive" result, where a query barcode is matched to a reference barcode despite significant divergence, is a frequent consequence of using the BLAST algorithm by itself [33]. For example, the query dataset used here contained five monobasic genera. For these barcodes the only possible result for a genus assignment using "best match" are "false-positive". These errors can be avoided by using the modified assignment criterion, "best close match". With "best close match" the best-matching reference barcode is identified, but the query is only assigned the taxon name of that barcode if the barcode is sufficiently similar (i.e. below a threshold). Otherwise, the query remains unassigned (i.e. "ambiguous"). In our case, the threshold value can be selected by plotting the number of "true-positives" and "false-positives" against the K2P distance from the query to the "best match". We then determine a threshold that maximizes the number of "true-positives" while minimizing the number of "false-positives". It remains unclear why one would expect that there should be a common threshold across taxonomic groups of the same rank or how this could be implemented in a real-life scenario. Many studies have shown a universal threshold of genetic distance to distinguish taxa cannot be determined [10]. However, in the absence of better strategies, this method at least provides a rigorously derived threshold value [25].

### Library species completeness

Based on their study of species in one family of Diptera, Ekrem et al. [9] concluded that assigning a barcode record to the correct genus or species-group was unlikely unless a "near perfect" match is present in the reference library with the further prediction that a "comprehensive" library is also essential for accurate assignment to family or even order. Furthermore, Ball and Armstrong [4] suggested that the failure of a lymantriine barcode to group with other members of its subfamily was attributable to low taxon sampling in their reference library (also see [5,34]). Considering that growth of the DNA barcode library will take time, a key issue concerns the effect of completeness of the reference library on the accuracy of higher taxon assignments. By using a global and comprehensive barcode reference library of considerable phylogenetic breadth (86% of known species in the family), the Sphingidae, we addressed this uncertainty through simulating different levels of species completeness of the reference library and examining the effect on assignment accuracy.

## Methods

### Query dataset, 100% reference library and sub-libraries

Using barcode records assembled as part of the global barcoding campaign on Sphingidae [15], we selected one barcode from each species to act as a reference barcode for that taxon. Reference barcodes were available for 1088 of the 1270 described species listed in Kitching and Cadiou [16] and for an additional seven Costa Rican species described or revalidated since 2000 (= 1095 sphingid species). Barcode sequences were selected to maximize length and quality and ranged from 267-658 bp, with 77% being 658 bp and 93% > 600 bp. The sample comprised 200 genera with all the currently recognised tribes and subfamilies (Figure 1A) represented. Three saturniid barcodes (*Arsenura drucei, Lonomia electra, Periga cluacina*) were also included as this family represents the putative sister family to the Sphingidae [35] taking the full reference library to 1098 barcodes (see additional file 1: Full reference library).

Barcodes from 118 sphingid species collected in Area de Conservacion Guanacaste, northwestern Costa Rica, were used as query barcodes (see additional file 2: Query dataset). DNA was extracted following automated protocols [36] and the DNA barcode amplified and sequenced [37]. These Costa Rican sphingids comprised a well-documented [38,39], diverse subset of the family, with each of the tribes and subfamilies represented among 29 genera. All the queries were correctly assigned to species when using the full reference library and a "best match" assignment criterion.

For the purposes of this study the following were considered libraries of 100% completeness: for genus assignment attempts, the representative from the same species as that of the query was the only barcode removed from the reference library; for tribe and subfamily assignment attempts, the barcodes from all the representatives of species in the genus of the query were removed from the reference library. All contribal genera were not removed in the case of subfamily tests, due to the increased level of uncertainty regarding naturalness of these taxa.

We subsequently created sub-libraries from the full reference library with different levels of species completeness. In an approach termed here "random sampling" barcodes were chosen at random to construct sub-libraries comprising 10, 20, 30, 40, 50, 60, 70, 80 and 90% of the full reference library. Sub-sampling at each species richness level was repeated 30 times. A different approach termed here "constrained sampling" limited the random selection of species to ensure a minimum of one species per genus in the sub-library. This approach was reiterated to construct sub-libraries comprising 20, 30, 40, 50, 60, 70, 80 and 90% of the full reference library and was repeated 30 times at each species completeness level. For the sub-libraries as with the 100% library, for genus assignment attempts, we removed the reference barcode for the species of the query from the sub-libraries. For tribe and subfamily assignment attempts we removed the reference barcodes for the genus of the query.

### Query assignment criteria

In each assignment attempt we allowed two possible outcomes: (i) A "positive" assignment (i.e. the query was assigned to a taxon) or (ii) An "ambiguous" assignment (i.e. the query was not assigned to a taxon). A "positive" assignment was either true (TP) - it matched with the morphology-based identification, or false (FP) - it disagreed with the morphology-based identification [40]. An "ambiguous" assignment was either true (TA) - the true taxon based on morphology was not represented in the reference library/sub-library (by at least two barcodes for "strict" criteria (Table 1)), or false (FA) - the true taxon based on morphology was represented in the reference library/sub-library (by at least two barcodes for "strict" criteria (Table 1)) [40].

The requirements for a "positive" assignment depend on the different criteria employed as detailed in Table 1. Note, the number of "potential TP" will not always be equal to 118 (i.e. the number of queries) because the taxon of the query may not be present in the sub-library. For example, the number of "potential TP" at the genus level with the 100% library and the "liberal" criterion is 113, due to 5 queries being members of monobasic genera.

We developed software in C++ to automatically construct sub-libraries, perform assignments according to four tree-based criteria and evaluate assignment success. The main tool took as input the queries, the outgroups, the complete reference library (all in fasta format), the sampling strategy, and an integer (X) indicating the percentage of the reference library to sampled. The software automated the analytical process as follows:

For each query:

For each replication:

Remove query species (or genus) from reference library.

Randomly select × percent of reference library without replacement according to input sampling strategy.

Combine query, outgroups, sampled reference library into a single file.

Construct NJ tree from file using Clustal W v.2 [41].

For each of four criteria:

Read tree, assign query a taxon or not according to criterion.

Evaluate accuracy of assignment (true or false).

**Figure 2:**
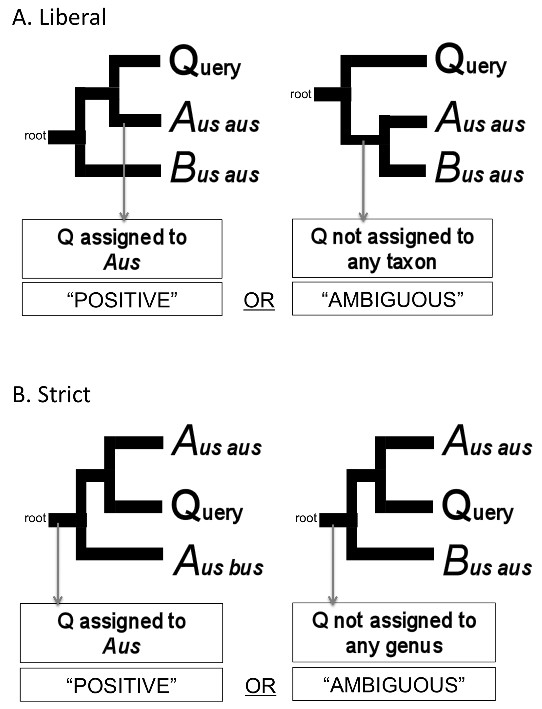
**Visualisation of two tree-based assignment criteria**. The distinction between a "positive" and an "ambiguous" assignment and how the assignment is achieved - based on the location of the query on the tree. A). "Liberal" - When the query barcode (Q) was sister to (*Aus*, Q) a clade comprising members of a single taxon, *Aus*, the assignment was that of the taxon in the clade, *Aus *(i.e. "positive"). When Q is sister to a clade comprised of multiple taxa, *Aus *and *Bus*, the assignment was "ambiguous". B). "Strict" - When the query sequence (Q) was nested within a clade comprising of members of a single taxon ((*Aus*, Q)*Aus*) the assignment was that of the taxon in the clade, *Aus *(i.e. "positive"). When Q is nested within a clade of multiple taxa ((*Aus*, Q)*Bus*)) the assignment was "ambiguous".

The four tree-based methods were "liberal" (Figure 2A) [40], "strict" (Figure 2B) [25,40], "liberal & exclusive" and "strict & exclusive". We also performed "best match" for all taxon assignments and "best match" and "best close match" for assignment to genus (with the randomly sampled library) where assignment was based only on the most similar reference library barcode (Table 1). For "best match" only a "positive" assignment is possible (i.e. the assignment is TP or FP) (Table 1). For "best close match" the query was assigned to the taxon of the most similar library barcode based on K2P distance, provided it was within a certain threshold. If there were no barcodes in the library within the threshold, the assignment was "ambiguous". In order to select a threshold we looked at the results of the "best match" criterion and plotted the number of "true-positives" and "false-positives" against the K2P distance from the query to the "best match". The distance that maximized the number of TP (which in our case also corresponded to the distance with the lowest proportion of FP) was selected as the threshold.

Measures of accuracy were calculated as follows: 1. Precision, the fraction of barcodes placed in a taxon that belongs there, TP/(TP+FP); and 2. Overall Accuracy, the proportion of barcodes placed without any error, (TP+TA)/(TP+FP+TA+FA) [33]. Note, for 'best match" due to the absence of the "ambiguous" category overall accuracy equals precision. The results are discussed below in terms of these measures.

## Results

The results of all the experiments are provided in additional file 3: Results of all experiments.

### Correct assignments to genus, tribe and subfamily (100% library)

The overall accuracy of assignment to genus was 0.83 using the "liberal" and 0.75 using the "strict" criterion. The precision of assignment to genus was 0.86 using the "liberal" and 0.98 using the "strict" criterion. A number of query species were consistently assigned to the wrong genus across all analyses resulting in FP. Even though these FP were technically incorrect assignments Table 2 details how in many cases the assignments made some sense considering the taxonomic structure and phylogeny of the family. These included four species in monobasic genera: *Pachylioides resumens, Phryxus caicus, Pseudosphinx tetrio*, and *Neococytius cluentius*, for which the only possible outcomes were FP or TA, since a query belonging to a monobasic genera cannot be a TP. A second group of FP were query barcodes (*Madoryx plutonius, Manduca albiplaga, Pachylia darceta *and *Pachylia ficus*) assigned to monobasic genera in the reference library (see Table 2). Queries belonged to species not present in the reference library, monobasic genera have only a single species that was present in the library, therefore, this group could more correctly be interpreted as TA or FA assignments. Two FP, *Xylophanes godmani *and *Xylophanes turbata*, were queries from an exceptionally species-rich genus (104 species globally). The overall accuracy of assignment to genus in this study was similar to that reported by Elias et al. [24] who found 69-81% of their Ithomiinae queries were assigned to the correct genus using tree-based criteria.

**Table 2:** False-positive assignments at the genus level

Query Sequence	Assignment	Notes
*Eupyrrhoglossum sagra *(2)	*Aellopos *(5)	*Aellopos *and *Eupyrrhoglossum *are most likely a sister pair [17]. *Eupyrrhoglossum *differs from *Aellopos *only in forewing veins Rs3 and Rs4 remaining separate apically and the phallus lacking spines on the right side (Kitching, personal communication).

*Madoryx plutonius *(4)	*Pseudosphinx *(1)	*Pseudosphinx *was close to *Madoryx *on the Kawahara et al. [17] phylogeny and both genera belong to the same tribe (Dilophonotini). *Pseudosphinx *is very close to *Isognathus*; indeed, it could be argued it is just an oversized *Isognathus *without yellow in the hindwing (Kitching, personal communication).

*Madoryx oiclus *(4)	*Hemeroplanes *(4)	*Hemeroplanes *was close to *Madoryx *on the Kawahara et al. [17] phylogeny and are considered a sister pair (Kitching, personal communication).

*Manduca albiplaga *(58)	*Apocalypsis *(1)	*Apocalypsis *and *M. albiplaga *were not sampled by Kawahara et al. [17], however, *Apocalypsis *was mentioned as an oriental genus expected to fall near the base of the Acherontini/Sphingini clade which included a paraphyletic *Manduca*.

*Neococytius cluentius *(1)	*Amphimoea *(1)	*Amphimoea *was not sampled by Kawahara et al. [17] but was placed by Kitching [49] as the sister to *Neococytius *+ *Cocytius*.

*Pachylia darceta *(3)	*Pachylioides *(1)	*Pachylioides *and *Pachylia *were not closely related on the Kawahara et al. [17] phylogeny, however, Rothschild and Jordan [50] noted a closer morphological similarity of *darceta *to *resumens*, both then being in *Pachylia*, than to the other two *Pachylia, ficus *and *syces*. Conversely, the larvae (e.g. direction of stripes) suggests a closer link between *darceta *and *ficus + syces *(Kitching, personal communication).

*Pachylia ficus *(3)	*Kloneus *(1)	*P. ficus *and *Kloneus *were sister taxa on the Kawahara et al. [17] phylogeny, and *Kloneus *is considered just a *Pachylia *with a crenulated forewing outer edge (Kitching, personal communication).

*Pachylia syces *(3)	*Phylloxiphia *(10)	*Phylloxiphia*, an African genus not sampled by Kawahara et al. [17], is part of the *Clanis *group of Smerinthinae and a long way removed from *P. syces *(Kitching, personal communication).

*Pachylioides resumens *(1)	*Pachylia *(3)	*Pachylioides *and *Pachylia *were not closely related on the Kawahara et al. [17] phylogeny, but were associated by Kitching and Cadiou [16].

*Phryxus caicus *(1)	*Erinnyis *(10	Not included by Kawahara et al. [17] but linked by Kitching and Cadiou [16] with *Phryxus *considered just a highly divergent *Erinnyis*.

*Pseudosphinx tetrio *(1)	*Madoryx *(4)	*Pseudosphinx *and *Madoryx *were reciprocally mis-assigned. See above.

*Xylophanes godmani *(80)	*Theretra *(38)	*X.godmani *was not sampled by Kawahara et al. [17] but *Xylophanes *and *Theretra *are members of the same tribe (Choericampina) and were suggested to be closely related by Hunsdoefer et al. [51] based on their mtDNA phylogeny.

*Xylophanes turbata *(80)	*Chaerocina *(3)	*X. turbata *was not sampled by Kawahara et al. [17], but the unexpected placement of *Chaerocina*, close to *Xylophanes*, was observed on their phylogeny.

Species and genus names are followed by a number in brackets which gives the number of species in the genus in the 100% reference library, e.g. *Neococytius cluentius *(1) is the sole member of *Neococytius *in the 100% reference library.

**Figure 3:**
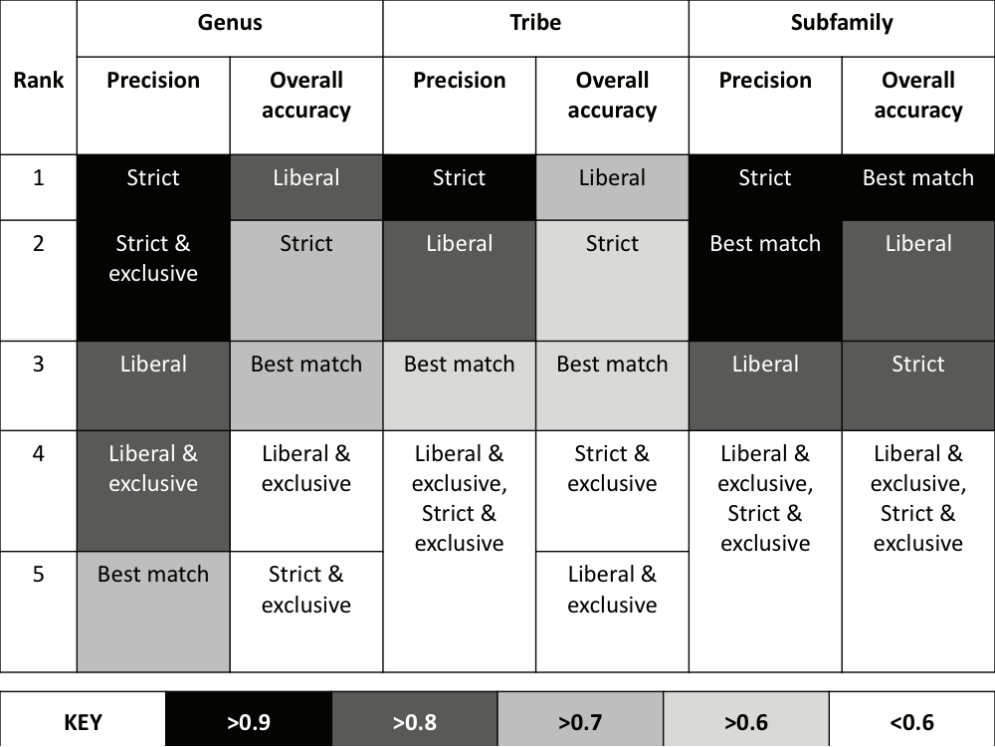
**Rankings of assignment criteria based on overall accuracy and precision**. Ranking of assignment criteria, four tree-based ("liberal", strict", "liberal & exclusive", "strict & exclusive") and one direct sequence comparison ("best-match") for assignments across three taxonomic levels (genus, tribe, subfamily) using a library with 100% of available species.

Overall accuracy of assignment to tribe was 0.75 using the "liberal" and 0.66 using the "strict" criterion (Figure 3). Precision of assignment to tribe was 0.81 using the "liberal" and 0.95 using the "strict" criterion. Many of the query barcodes placed in the wrong tribe belonged to genera that are positioned as paraphyletic or polyphyletic with respect to their current tribal designations, according to recent phylogenetic study (e.g. *Agrius, Aleuron, Cautethia, Cocytius, Enyo, Eumorpha, Pachygonidia *[17]), or were on long branches in a basal position (*Pachylia*) within their tribe. An instructive example is *Eumorpha*, a genus currently placed in the tribe Philampelini. Query barcodes belonging to *Eumorpha *were assigned to tribe Macroglossini. This is consistent with the placement of *Eumorpha *(+*Enyo*) as sister to a clade comprised of Macroglossini on the phylogeny of Kawahara et al. [17].

Overall accuracy of assignment to subfamily was 0.90 using the "liberal" and 0.84 using the "strict" criterion with "best match" having the highest overall accuracy for this taxonomic level (0.92) (Figure 3). Precision of assignment to subfamily was 0.83 using the "liberal" and 0.96 using the "strict" criterion.

### Success of tree-based assignment criteria

**Figure 4:**
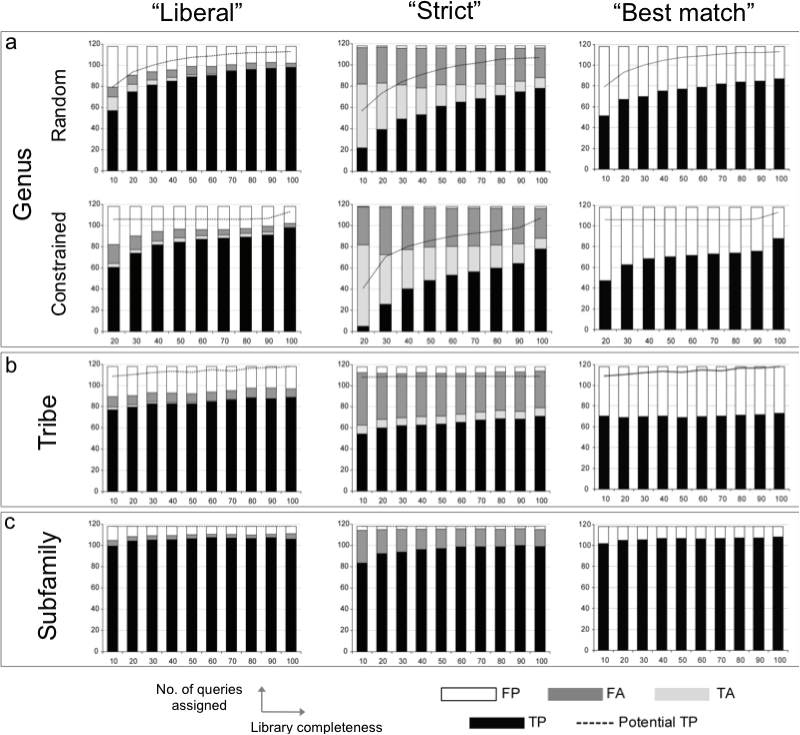
**Taxonomic assignment success for "liberal", "strict" and "best match"**. DNA barcode higher taxonomic assignment success across three taxonomic levels (genus, tribe, subfamily) using two tree-based criteria ("liberal", "strict") and one direct sequence comparison criterion ("best match") for 118 query species, with reference sub-libraries of varying species completeness (10-100% of available species).

**Figure 5:**
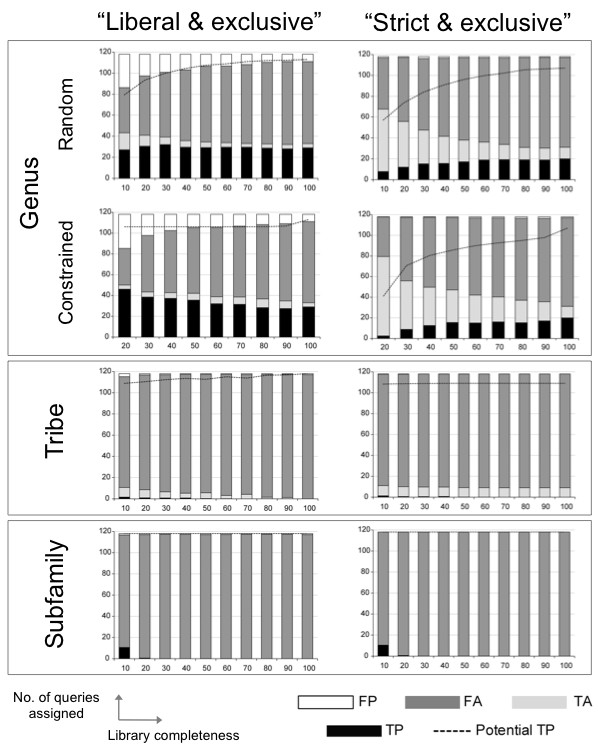
**Taxonomic assignment success for exclusivity criteria**. DNA barcode higher taxonomic assignment success across three taxonomic levels (genus, tribe, subfamily) using two tree-based criteria requiring exclusivity of taxa ("liberal & exclusive", "strict & exclusive") for 118 query species, with reference sub-libraries of varying species completeness (10-100% of available species).

Considering Figures 4 and 5, it is clear that different criteria produced contrasting results. For example, "liberal" was frequently the highest scoring criteria in terms of overall accuracy (Figure 3), but performed less well in terms of precision with an average of 18% of assignments to genus being FP (Figure 4). "Strict" had lower overall accuracy across all sub-libraries, but higher precision with an average of only 2% of assignments to genus being FP (Figure 4).

The criteria requiring exclusivity resulted in an overwhelming number of FA assignments (Figure 5) and produced very low overall accuracy and precision despite their lower incidence of FP (Figure 5). Note that the success rate for criteria without the exclusivity requirement are higher, because they did not require "monophyly"; i.e. queries can be assigned on trees with congeneric (or contribal and subfamilial) barcodes found in two different "clades" as long as the rules of the criterion are met.

### Success of sequence comparison assignment criteria

Success under "best match" was similar to "strict" at the tribe level but very similar to "liberal" at the subfamily level (Figure 4), where it actually had the highest overall accuracy but was still behind the "strict" criteria in terms of precision (Figure 3).

**Figure 6:**
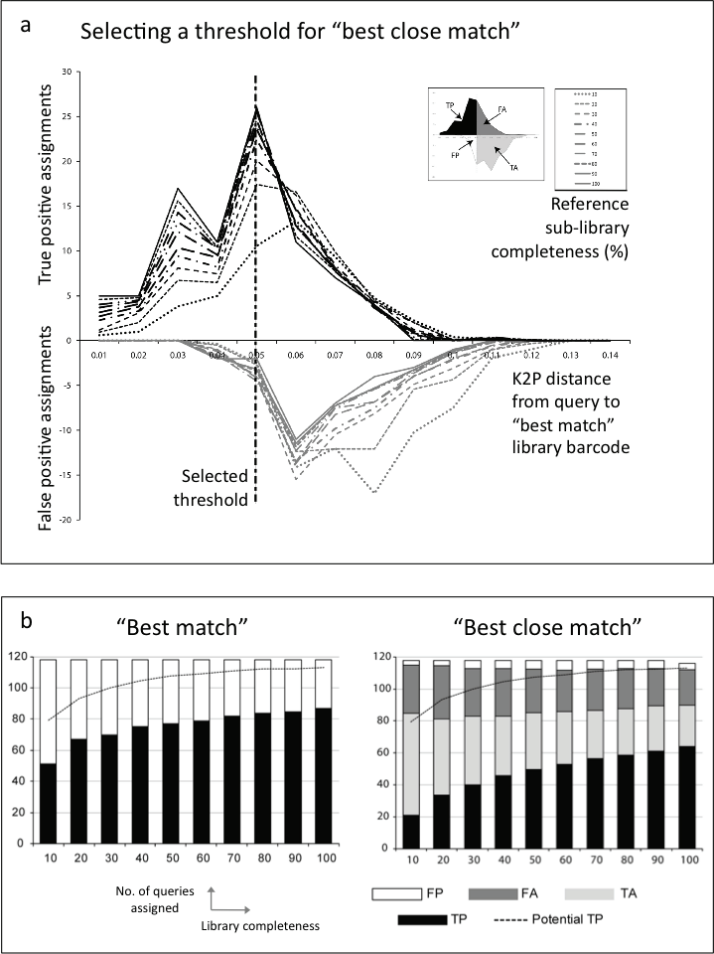
**The "best match" criterion used for genus assignments**. a) Selecting a threshold for the "best close match" criterion. We looked at the results of the "best match" criterion and plotted the number of assignments (y axis) against the K2P distance (x axis) between the queries and the "best match" reference library barcodes ("true positives" as positive values above the × axis and "false positives" below the × axis). The distance that maximized the number of TP (which in our case also corresponded to the distance with the lowest proportion of FP) was selected as the threshold based on visual inspection of the histogram (i.e. 0.05) and is indicated by the dashed vertical line. The small inset chart is a cartoon showing how each section of the larger chart corresponds to each of the four "best close match" assignment outcomes (TP, FP, TA and FA) depending on the selection of a threshold i.e. placement of a vertical line. b) DNA barcode genus assignment success based on two sequence direct comparison criteria ("best match", "best close match") for 118 query species, with reference sub-libraries of varying species completeness (10-100% of available species selected randomly).

In order to be able to use "best close match", we first determined the optimal threshold to be 0.05 K2P distance (Figure 6) and this value was used to decide whether a query had a close enough barcode match be given a "positive" assignment. "Best close match" successfully reduced the high number of FP seen with "best match" (Figure 6), but, like the "strict" criterion resulted in a large number of FA. Success under "best close match" was very similar to "strict" but it produced a much lower number of TP with the larger sub-libraries (Figure 6).

### Effect of library completeness

**Figure 7:**
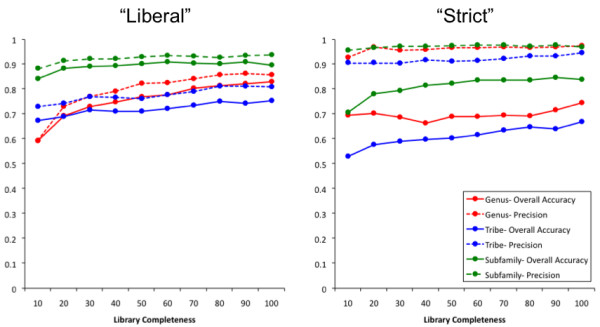
**Overall accuracy and precision across library completeness**. Average overall accuracy and precision scores across DNA barcode libraries of varying completeness (10-100% of available species) for two tree-based assignment criteria, "liberal" and "strict".

The "liberal" and "strict" criteria were generally the highest-scoring criteria in terms of overall accuracy and precision across all taxonomic levels and all sub-libraries (Figure 3 and 4). An exception was the high precision observed for the "strict & exclusive" criterion that was matched by extremely low overall accuracy (Figure 5). Precision was consistently high for the "strict" criterion (> 0.90) for all sub-libraries and for assignments to all taxonomic levels. Precision was lower using "liberal" but conversely overall accuracy was higher (Figure 7).

The effect of library completeness was visible in assignment to genus using "liberal" with overall accuracy increasing from 0.59 with the 10% sub-library to 0.83 with the 100% library (Figure 7). Using "strict" however, overall accuracy although generally lower was relatively stable regardless of library completeness, increasing only 0.06 between the 10% and 100% libraries. The opposite pattern was seen in overall accuracy of assignments to tribe and subfamily with "liberal" being more stable across sub-libraries, and "strict" being more variable (Figure 7).

Results for assignment to genus using random versus constrained sampling of sub-libraries were very close in terms of overall accuracy, with constrained having slightly lower overall accuracy across all completeness levels (Figure 4a). Conversely, constrained sub-libraries resulted in assignments with slightly higher precision across all completeness levels.

## Discussion

We present the results from an in-depth study of higher taxon assignment using DNA barcoding. The reader of DNA barcode literature may be surprised by the assignment accuracy reported here, values that may contrast with the expectation of authors like Ekrem et al. [9]. This may be explained largely by differences in study design. Our experimental design measures the relative precision and overall accuracy of different assignment criteria across reference libraries of different levels of completeness and structure. No single assignment criterion was superior across the range of taxonomic scenarios examined and there was often a conflict between overall accuracy and precision. Our results discussed below, together with implications for criterion selection, indicate a clear requirement for species to be in taxa that are well-differentiated clades to maximize the number of correct assignments. Whether these success rates are high enough to be useful remains a judgment call for the end-user.

### Assessing barcoding accuracy with taxonomic classifications

In this study we have presented simplified examples where the species of the query barcode is missing from reference libraries (and the entire genus for assignments to tribe and subfamily) to ensure we were solely addressing the question of assigning the query to the next least inclusive taxon. By excluding the possibility of a species (or genus) match, which would effectively provide the higher taxonomy of the query, this study was a rigorous test of the effect of assignment criteria and species completeness of the reference library on higher taxon assignments. The arbiter of success was necessarily a classification [16] that is already considered "out of date" [17,42]. As such, a pertinent issue to DNA barcoding success is taxonomy/species tree incongruence as well as species tree/gene tree incongruence [43]. This is especially the case for the large species-rich genera e.g. *Xylophanes, Manduca*, where generic boundaries may need to be revisited [17] (Table 2). The effect of using an "old" classification was perhaps particularly apparent when considering the results of the tribe experiments, although adoption of a new classification did not appear to improve assignment accuracy (data not presented). FP at the tribe and subfamily level often reflected new knowledge of Sphingidae phylogeny [17], and therefore reflect real phylogenetic signal among barcode sequences.

The Sphingidae has received a relatively extensive treatment by taxonomists. This raises a concern for other less-well-studied groups; how accurately can barcoding be expected to assign queries to taxa that are most likely not natural [24,44,45]? While this study provided few examples where the barcode assignment was clearly at odds with current taxonomic understanding, it would be much more difficult to assess in other moth families. Despite most systematists adhering to cladistics since Hennig [46], many "good" Lepidoptera taxa, including those within Sphingidae [17], lack reliable (private) morphological synapomorphies which would enable rapid assignment of species to higher taxa. It is difficult to assess how our results would compare with morphological assignment accuracy by a non-specialist. However, it is clear that even a specialist taxonomist would have difficulty in assigning an egg to a genus, while DNA barcoding can be used with any tissue sample from any life stage. There are groups of species e.g. from Microlepidoptera, Pyralidae, that are far more difficult to assign morphologically to taxa and lack of morphological synapomorphies may reflect the instability of the current classification. The results presented here echo the relative stability of subfamilial and generic taxa compared to the tribes [16,17] suggesting the real challenge in taxonomy is to build new, robust phylogenies, and ensure that these are reflected in the classification.

Another challenge highlighted by our study is the lack of equivalency of taxonomic ranks [47] in terms of genetic distance [48]. This is clear through our inability to increase success of sequence comparison criteria through the use of "best close match". An optimal threshold will always be taxon specific and even within a relatively small group a universal threshold is unlikely to be effective. Avise and John [48] proposed a temporal scheme to standardize taxonomic ranks. However, an obvious objection is that the scheme would require significant revisions of all groups. Instead of speeding up taxonomic work, to be effective, large-scale employment of a distance-based assignment criterion would have to start with the redefinition of most taxa. Like Kelly et al. [33], we furthermore found that tree-based criteria outperform the direct sequence comparison methods, thereby rendering threshold values for "taxon-level" divergences unnecessary.

### Effect of library structure and completeness

When the query's taxon was not in the reference library, only "strict" was relatively immune to FP, and consequently was the best scoring criterion in terms of precision. Given this ability to limit FP, "strict" was also the criterion for which overall accuracy was least affected by reference library completeness.

It seems intuitive that "best match" would perform better in libraries where taxon matches are always available. However, in real-life, it is impossible to know whether a query is from a "new" taxon or from a taxon that is already represented in the reference library. Considering the problems with direct sequence comparison methods, relying solely on distances, we do not believe they are promising tools although arguably these are the most practical. Interestingly, "best match" had highest overall accuracy, beating tree-based criteria at the highest taxonomic level investigated in this study - subfamily.

We tested criteria that allow for "ambiguous" assignments and found library completeness had a weak effect and high overall accuracy and precision was seen at low completeness. Our comparison of constrained and randomly selected reference sub-libraries showed that accuracy is not compromised by the absence of taxa in the reference library. We found that whether the library was incomplete or all species were present in the library, the criteria selected to provide an assignment was still a factor determining success.

### Strategies for higher taxonomic level assignment

Techniques for assigning sequences to a higher taxon are still in their infancy, but new methods are appearing more frequently (e.g. CAOS [28]). Based on our results, we suggest a conservative approach that initially uses a "strict" tree-based criterion in large-scale assignment systems. Although a large number of queries would remain ambiguous due to the more conservative nature of the criterion, we nevertheless consider this result with its higher precision to be preferable to an assignment criterion like "best match", which yields marginally more TP but also a large number of FP. Criteria requiring exclusivity were the most conservative, but given their very low overall accuracy and precision they would probably only be justifiable for forensic purposes [19].

Tree-based criteria could be easily incorporated into the current library set-up (BOLD), by providing higher taxonomy alongside the species name attached to barcodes on a Taxon-ID tree. The current approach offered by BOLD uses a similarity search to collect the top 100 hits in the reference library and then constructs a NJ tree to allow the attachment of a query barcode to this 100 best backbone tree [3]. From this tree an attempt can be made to assign the query to genus using "strict". However, if no "positive" genus assignment can be made, an attempt could be made at assignment to tribe, etc. Alternatively, an assignment can be attempted to a taxonomic level determined by the taxonomic sufficiency requirements of the investigators. For example, water monitoring using invertebrate diversity indices may only require samples be identified to family to be useful. Some could argue that heuristic taxonomic groupings (OTUs) based on barcodes are better than no taxonomic hypothesis at all, and certainly superior to heuristic morphology-based equivalents, being rooted in a standardised, objective, consistently coded character set.

## Conclusions

Our empirical test of higher taxonomic assignments reveals that a tree-based assignment system would successfully assign most queries to a higher taxon at some level. A conservative approach using the "strict" tree-based method should be used initially in large-scale identification systems. The failures we observed do not make us question the usefulness of barcode libraries for generic and suprageneric assignments. They indicate imperfect taxonomy and suggest that the barcodes themselves could aid our ability to revise non-natural taxa. An advantage of any DNA-based system is that the data are readily available for further analysis with alternative models or approaches. Discounting DNA barcoding as a tool for providing taxonomic assignments because the library is not yet complete is pusillanimous.

## Authors' contributions

JJW, RR, JS, PDNH conceived of the project and designed experiments. JJW, DHJ, WH, MH assembled the query dataset. RR, IJK, JH assembled the sphingid reference library and made it available for this study. JJW, RR, JS performed the experiments and analyzed the data. JJW, RR, JS drafted the manuscript. DHJ, WH, MH, IJK, PDNH assisted in writing the manuscript. All authors read and approved the final manuscript.

## Supplementary Material

Additional file 1 **Full reference library**. Excel worksheet containing a list of COI sequences used in the full reference library in this study, including the species name attached to the barcode and BOLD (http://www.barcodinglife.org) Sample IDs and Process IDs. Click here for file

Additional file 2 **Query dataset**. Excel worksheet containing a list of COI sequences used as the query dataset in this study, including the species name attached to the barcode and BOLD (http://www.barcodinglife.org) Sample IDs and Process IDs. Click here for file

Additional file 3 **Results of all experiments**. Excel worksheet containing the summarized results of all the experiments performed. Click here for file
